# Revisiting giant virus-host dynamics in brown algae: old stories and new perspectives

**DOI:** 10.1038/s44318-025-00667-y

**Published:** 2026-01-03

**Authors:** Carole Duchêne, Liping Wang, Susana M Coelho

**Affiliations:** Max Planck Institute for Biology Tübingen, 72076 Tübingen, Germany

**Keywords:** Brown Algae, Viruses, Nucleocytoviricota, Evolution & Ecology, Microbiology, Virology & Host Pathogen Interaction

## Abstract

The recent discovery of widespread giant virus sequences integrated into the genomes of diverse eukaryotes, and in particular marine lineages, has reignited interest in the molecular mechanisms underlying giant virus–host interactions. The brown alga *Ectocarpus* represents a compelling and historically rich model for such studies. As early as the 1970s, it was used to investigate latent infections by giant double-stranded DNA viruses, with elegant classical genetics and electron microscopy approaches revealing key aspects of virus–host life cycle coordination. However, progress was limited by the lack of molecular and genomic tools. In this review, we revisit these foundational studies through the lens of recent technological advances, including the development of genetic and genomic resources for brown algae. These tools now enable mechanistic insights into giant viral integration, latency, activation and host response. We highlight how *Ectocarpus* and related systems can illuminate both the evolutionary and ecological dimensions of virus–host dynamics, with a particular emphasis on the molecular and genetic mechanisms that mediate these complex interactions.

## Abstract (web summary)

Ectocarpus re-emerges as a powerful system for dissecting the ecology and evolution of giant virus–host dynamics.

## Graphical abstract

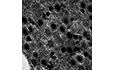

## Introduction

**Figure 1:**
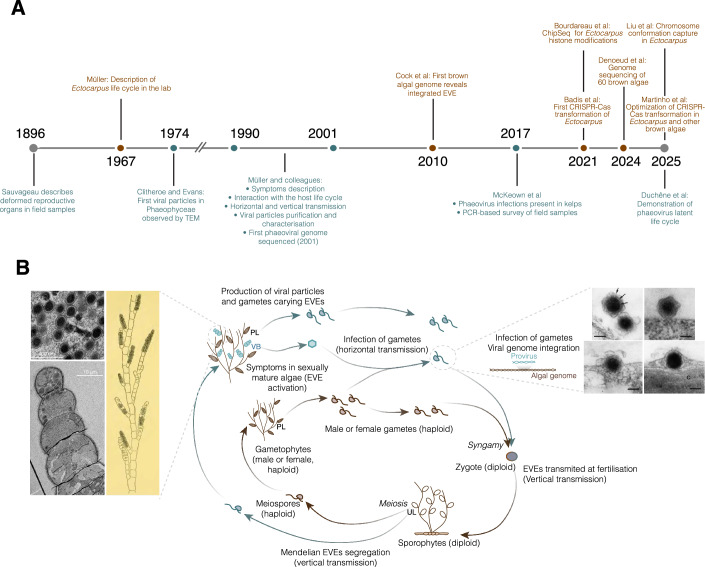
The *Ectocarpus*–phaeovirus system: from historical observations to modern tools. (**A**) Timeline summarizing the history of *Ectocarpus*–phaeovirus research. Top: Important milestones in *Ectocarpus* research (in brown). Bottom: Discovery and foundation work on phaeoviruses (in blue). (**B**) Simplified diagram of *Ectocarpus* life cycle showing the stages where viral activation occurs. The brown inner arrows depict the life cycle for *Ectocarpus* with no virus infection, whereas the blue outer arrows illustrate how the virus manipulates the host life cycle. The algal sporophyte (diploid) produces meiospores via meiosis in the unilocular sporangia (UL). The (haploid) meiospores inherit EVEs present in the sporophyte genome in a Mendelian manner (horizontal transmission of the latent EVE). They develop as male and female gametophytes, which in turn produce gametes by mitosis in reproductive structures called plurilocular sporangia (PL). If the meiospore has inherited an active EVE, it can develop viral symptoms in plurilocular sporangia in the form of viral bladders (VB). Theses viral bladders were first described in 1896 by Sauvageau (see drawing on the left, from Sauvageau (1896)), and consist of swollen cells filled with viral particles (Transmission Electron Microscopy (TEM) images on the left). Male and female gametophytes release male and female gametes, while actively infected individual release viral particles. These particles can infect naïve, cell-wall-free, swimming gametes, by attaching to their cell surface (Right; TEM images from (Maier et al, 2002). The viral genome is then released into the gametes and transferred to the nucleus, where it integrates into the algal genome. Note that gametophytes infected by the virus may also produce healthy gametes, that carry the integrated EVE in their genomes. These gametes then fuse with gametes from the other sex, giving rise to a new diploid zygote, transmitting the latent virus to the next generation.

Viruses are now recognized as the most abundant biological entities in marine habitats, a finding that has reshaped our view of ocean ecosystems and their complexity (Suttle, 2005). Among them, giant viruses of the phylum *Nucleocytoviricota* have emerged as major players in eukaryotic biology, with an expanding diversity that continues to redefine virus–host relationships across the tree of life (Forterre and Gaïa, 2016). Marine eukaryotes serve as important hosts for these viruses, and their infections can strongly influence microbe population dynamics and phenomena such as algal blooms (Vincent and Vardi, 2023). In particular, viral lysis of phytoplankton not only terminates blooms by directly reducing host cell abundance but also releases dissolved organic matter that fuels microbial loop processes and alters nutrient cycling (Hevroni et al, 2023). In some cases, viral activity can control the timing, magnitude, and succession of blooms, shaping community structure at ecosystem scales. Moreover, interactions between viruses, algal hosts, and other microbial partners can have cascading effects on biogeochemical fluxes, including carbon export and oxygen production in the ocean (Mojica and Brussaard, 2025). Collectively, these observations highlight marine viruses as key regulators of ocean ecosystem dynamics and biogeochemical fluxes. Within this broader framework, phaeoviruses, large double-stranded DNA viruses of the phylum *Nucleocytoviricota*, represent a particularly intriguing case within marine virus–host interactions. These viruses infect multicellular brown algae (Box 1), establishing persistent infections that can be transmitted through the host’s reproductive life cycle. Early studies, particularly those conducted in the 1990s, suggested a latent life cycle with integration of viral DNA into the host genome and controlled expression of viral genes (Fig. 1). Classical approaches, combining genetics, cytology, and electron microscopy provided key insights into how these viruses maintain long-term infections without immediately lysing their hosts. These foundational studies established a strong conceptual framework, demonstrating that these viruses could serve as models for understanding virus–host coordination in complex multicellular eukaryotes. Despite this early promise, progress in the field stalled over the past decades. The lack of molecular and genomic tools limited mechanistic studies, and consequently, the biology of phaeoviruses remained poorly understood relative to other giant viruses.

This situation is changing rapidly. Recent advances in brown algal genomics and molecular biology provide an opportunity to revisit earlier studies. With modern tools, we can now dissect the molecular mechanisms of phaeovirus integration, latency, activation, and host response, paving the way for a deeper understanding of how these viruses influence host biology and ecosystem dynamics. This review places these pioneering studies in the context of emerging molecular insights, highlighting the contributions of new tools for brown algae research, particularly in the model organism *Ectocarpus*.

Box 1 What are brown algae? Brown algae (Phaeophyceae) represent a highly diverse lineage of Stramenopiles that has independently evolved complex multicellularity. Their wide range of morphologies, life cycles, and reproductive strategies means that many species within this group have strong potential to serve as model systems for addressing fundamental biological questions. Established models include the fucoids, which have long been used for cell biology studies (Coelho et al, 2002; Coelho and Cock, 2020), and *Ectocarpus* sp., which is increasingly employed for molecular genetics and developmental research. More recently, kelps such as *Saccharina* and *Macrocystis* have emerged as promising systems for studying tissue differentiation, large-scale thallus morphogenesis, and ecological adaptations. Together, these species provide opportunities to investigate development, reproduction, and evolution across the brown algal lineage, while also offering insights relevant for biotechnology and sustainable resource applications.

Brown algae as comparative models for multicellularity and development. Brown algae (Phaeophyceae) are multicellular Stramenopiles, evolutionarily distinct from land plants, red algae, and opisthokonts. They display a broad range of morphologies, from simple filaments (*Ectocarpus* sp.) to large parenchymatous thalli with multiple cell types (kelps, Laminariales), and exhibit diverse reproductive strategies and sexual systems (Batista et al, 2024). Their independent evolution of complex multicellularity and unique developmental programs makes them valuable comparative models for understanding the origins and mechanisms of multicellularity, tissue differentiation, and life-cycle regulation. Established models include fucoids (*Fucus* sp.) for cell biology, *Ectocarpus* sp. for molecular genetics, and kelps for morphogenesis and ecological studies, providing complementary systems to study fundamental biological processes in a lineage distant from plants and animals.

**Figure:**
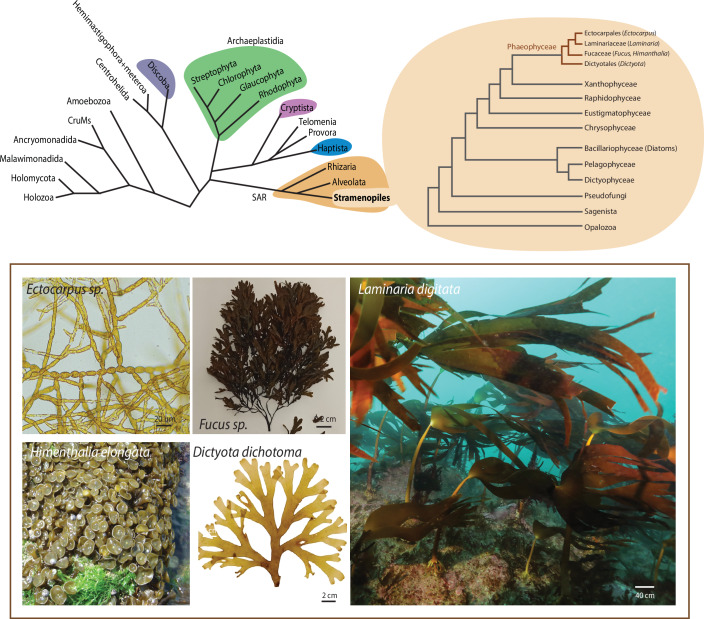
**Figure Box 1**. The diversity of Eukaryotic algae: schematic tree of Eukaryotes, adapted from Williamson et al, 2025, and expanded Stramenopile branch to illustrate the position of Phaeophyceae (adapted from Cho et al, 2024; Denoeud et al, 2024; Terpis et al, 2025). Lineages containing eukaryotic algae are highlighted in color. Photos kindly provided by Kenny Bogaert, Rémy Luthringer and Wilfried Thomas (CNRS).

## Giant viruses infecting algae

*Nucleocytoviricota*, formerly referred to as the nucleocytoplasmic large DNA viruses (NCLDV), constitute a distinct viral phylum of double-stranded DNA viruses with exceptionally large genomes, in some cases exceeding 2.5 Mb (Philippe et al, 2013; Fischer, 2016). Members of this group display remarkable host diversity, infecting organisms ranging from mammals to microalgae, and encompassing the vast phylogenetic breadth of protists. Notably, *Acanthamoeba*, the host from which the first giant virus (mimivirus) was isolated, has become emblematic in the study of this lineage (Raoult et al, 2004). In addition to these well-characterized systems, a multitude of yet-unidentified hosts are thought to sustain the extensive diversity of *Nucleocytoviricota* (Aylward et al, 2021; Schulz et al, 2022).

**Figure 2:**
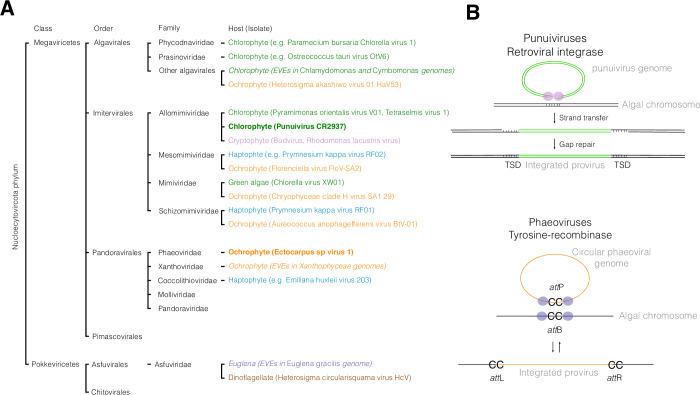
*Nucleocytoviricota* classification and mechanisms of integration. (**A**) Schematic tree of *Nucleocytoviricota* classification, highlighting viruses infecting algae. In italics are viruses known only from EVEs (Endogenous Viral Elements) in their host genome, while the bold highlight the two viral lineages for which a latent life cycle is demonstrated (phaeoviruses) or strongly suspected (punuiviruses). Tree was adapted from (Aylward et al, 2021; Koonin et al, 2024), with additional lineages from (Nagasaki et al, 2003; Ogata et al, 2009; Moniruzzaman et al, 2022; Sheng et al, 2022; Aylward et al, 2023; Thomy et al, 2024; Vieira et al, 2024; Byl et al, 2025; Jivaji et al, 2025). (**B**) Proposed mechanisms for integration of viruses with latent integrated life cycle. Punuiviruses integration takes place via retroviral integrases (see text), leaving Target Site Duplications (TSD), while phaeoviruses integrate via a tyrosine recombinase. For simplicity and to explain the formation of the TSD, only the retroviral mechanisms is represented as double-stranded DNA.

Historically, double-stranded DNA viruses infecting algae were classified within the *Phycodnaviridae* family, a grouping based primarily by pioneering studies of eukaryotic algae from both marine and freshwater environments (Wilson et al, 2009). Representative examples include the green alga *Chlorella*, targeted by chloroviruses such as Paramecium bursaria chlorella virus (PBCV), and brown algae (Phaeophyceae), which host phaeoviruses such as Ectocarpus siliculosus virus-1 (EsV-1). These viruses were initially considered to form a monophyletic order within *Nucleocytoviricota*. However, the identification of novel virus–host associations, coupled with insights from environmental meta-omics, has fundamentally challenged this view (Aylward et al, 2021). Indeed, recent phylogenetic analyses, including metagenome-assembled viral genomes, place algae-infecting viruses in different orders of *Nucleocytoviricota*: coccolithoviruses (infecting the haptophyte *Emiliana huxleii*) and phaeoviruses are part of the proposed ‘*Pandoravirales*’ order, prasinoviruses infecting green algae in the *Algavirales* order, while viruses infecting diverse algae such as the stramenopile *Aureococcus*, the green algae *Tetraselmis* and the haptophyte *Prymnesium kappa* are classified in different families of the *Imitervirales* (Aylward et al, 2021; Koonin et al, 2024) (Fig. 2). Beyond their genetic diversity, these viruses exert profound ecological impacts by modulating algal population dynamics, driving bloom termination, and shaping nutrient cycling and carbon export in aquatic ecosystems (Mojica and Brussaard, 2025). It is perhaps unsurprising that the diversity of viruses infecting algae mirrors the evolutionary breadth of their hosts, as algae represent a deeply divergent assemblage of eukaryotes with complex evolutionary histories. Notably, even within the same algal genus, distinct giant viruses from different clades have been shown to establish infections (e.g., Prymnesium kappa viruses and Haptolina ericina virus infected the same hosts (Johannessen et al, 2015)). A substantial fraction of viral clades, however, remain without identified hosts. Among more than 2500 *Nucleocytoviricota* genomes sequenced to date, only around 200 originate from cultivated isolates with known hosts (Schulz et al, 2022). Emerging methodologies are beginning to address this gap, including approaches based on co-occurrence and interaction networks (e.g., Endo et al, 2020), single-cell genomics (e.g., Needham et al, 2019), and more recently, single-cell transcriptomics (e.g., Fromm et al, 2024) and chromosome conformation capture (Hi-C) techniques (e.g., Bignaud et al, 2025). Together, these approaches hold promise for resolving previously unknown host–virus associations.

The viruses known to infect brown algae (Phaeophyceae) form a monophyletic lineage, the phaeoviruses, within the proposed order ‘*Pandoravirales*’. Their closest characterized relative is coccolithoviruses, e.g., the Emiliania huxleyi virus (EhuxV), a central model in marine virology (Ku et al, 2020; Kuhlisch et al, 2021; Joffe et al, 2024; Shaler et al, 2025). Despite their ecological and evolutionary significance, the biology of phaeoviruses remains poorly understood, with most available knowledge stemming from the pioneering studies of Dieter Müller and colleagues during the 1990s and early 2000s (Fig. 1A). Their unusual infection strategies and complex interactions with algal hosts (Müller et al, 1998; Schroeder, 2015) suggest that they represent an especially compelling system for future study of host–virus interactions and latent life cycles. Among them, EsV-1 has emerged as the best-studied representative, providing a foundation for our current understanding of phaeovirus biology.

### Historical foundations: the *Ectocarpus*-phaeovirus system

Viral symptoms in *Ectocarpus* were described almost simultaneously with the discovery of the alga itself. In the 1890s, as phycologists documented brown algal diversity across Europe, the French phycologist Camille Sauvageau reported abnormal reproductive structures and proposed that these abnormalities were caused by an unidentified parasite (Sauvageau, 1896) (Fig. 1A). Nearly 80 years later, virus-like particles were first visualized by electron microscopy in the reproductive organs of *Ectocarpus* (Clitheroe and Evans, 1974). Foundational work by Dieter Müller and colleagues in the 1990s subsequently elucidated key aspects of phaeovirus biology. In their initial descriptions, Müller and collaborators demonstrated that viral symptoms could be transmitted horizontally via infection of zoids (Müller et al, 1990). In addition to horizontal transmission, viral symptoms were also observed to persist through mitotic divisions in algal cultures, and to be inherited vertically throughout the algal life cycle, both at fertilization and during meiosis. Strikingly, the symptoms segregated in a Mendelian fashion, leading to the early hypothesis that the viral genome existed in a proviral form, closely associated with, or integrated into one of the host autosomes (Müller, 1991). Subsequent studies from the same laboratory (e.g., Delaroque et al, 1999; Delaroque and Boland, 2008), together with more recent work (Duchêne et al, 2025, see “The genomic era” section below), confirmed this hypothesis, establishing that the viral genome is maintained as an endogenous viral element (EVE) inserted into the algal genome.

*Ectocarpus* alternates between haploid (gametophyte) and diploid (sporophyte) stages, both of which develop from free-living multicellular stages (Fig. 1B). Infectious particles can be observed in cultures at specific developmental transitions, particularly during gametogenesis and sporogenesis. Environmental factors, such as temperature decreases (from standard growth conditions at 14 °C to 10 °C) increases the severity of viral symptoms, with a higher proportion of reproductive structures producing viral particles (Müller, 1991; Lanka et al, 1993). The released particles are capable of infecting naive *Ectocarpus* wall-less zoids (Müller et al, 1990; Klein et al, 1995, Fig. 1B). Upon infection, the virus attaches to the target cell using a yet unknown mechanism and releases its viral core into the cytosol. The viral core migrate to the nucleus, where the viral DNA integrates into the host genome, completing the infectious cycle (Maier et al, 2002).

The *Ectocarpus–*EsV-1 system exemplifies a lysogenic viral strategy: long-term maintenance as a heritable, largely “silent” EVE integrated into the algal genome, punctuated by environmentally or developmentally triggered reactivation that restores a lytic phase. At the time of its discovery, however, there was no direct proof linking the integrated viral element with either the appearance of symptoms nor the production of viral particles. Conclusive evidence for this connection only came to light more than 30 years later (Duchêne et al, 2025, see “The genomic era” section below).

In addition to documenting symptom transmission, Dieter Müller’s laboratory also characterized phaeovirus virions, investigating particle morphology and, notably, beginning to analyze individual viral proteins (e.g., GP1, VHK (Klein et al, 1995; Delaroque et al, 2000a; Delaroque et al, 2000b)) well before the advent of modern proteomics. The group further provided the first descriptions of the viral genome (Lanka et al, 1993; Lee et al, 1995), work that culminated in the full-length sequencing of the EsV-1 genome in 2001 (Delaroque et al, 2001). Importantly, Müller’s research was not confined to a single *Ectocarpus* strain in culture but extended across diverse *Ectocarpus* isolates and other brown algal species from different geographical origins, thereby demonstrating the global prevalence of these viruses in Phaeophyceae (Müller and Stache, 1992; Sengco et al, 1996). Comparable symptoms and associated virus-like particles were later reported in other brown algae (Kapp et al, 1997; McKeown et al, 2017), though never with the same level of detail achieved in the *Ectocarpus*–EsV-1 system, which subsequently emerged as the model for phaeovirus biology.

Following this pioneering phase, progress slowed considerably after the early 2000s, largely due to the absence of genetic tools for studying brown algal hosts, which severely constrained mechanistic research on phaeoviruses (see Fig. 1A). Only sporadic studies, primarily addressing ecological aspects, were published between 2000 and 2020 (e.g., McKeown et al, 2018; see ecology section below). The field was revitalized, however, with the discovery that phaeoviruses are not only globally distributed but also abundant as integrated elements within brown algal genomes (Denoeud et al, 2024), placing them back at the forefront of marine virology.

### The genomic era: hidden giant viruses in brown algal host genomes

Recent advances in sequencing technologies revealed the breadth and the ubiquity of viral elements integrated in eukaryotic genomes. With more and more (good quality) eukaryotic genomes becoming available, we are only starting to realize the diversity of *Nucleocytoviricota* and their hosts and how these interactions shaped the evolution of eukaryotes (Gallot-Lavallée and Blanc, 2017; Moniruzzaman et al, 2022; Zhao et al, 2023; Sarre et al, 2024; Jivaji et al, 2025). However, if the detection of EVEs in a genome can inform on past encounters between a host and its viruses, the physiological and evolutionary meaning of these insertions is not always evident.

The *Ectocarpus* genome, and the Phaeophyceae-phaeovirus system in general, exemplifies the relationship between viral insertion and viral symptoms, as well as the dynamics of these insertions across evolutionary times. The first *Ectocarpus* genome to be sequenced was found to host several virus pieces, including the insertion of a putatively full-length phaeovirus closely related to EsV-1 (Cock et al, 2010). However, this *Ectocarpus* strain (species 7, strain Ec32) is not the same strain as the one used in the historically studies on EsV-1, and never showed symptoms in culture. The *Ectocarpus* strain hosting EsV-1 was sequenced only later, along with other *Ectocarpus* strains showing symptoms, allowing the definitive demonstration that phaeoviral EVEs are reactivating to cause symptoms using classical and reverse genetics (Duchêne et al, 2025). These genomes host one or several active EVEs (i.e., able to cause symptoms), along with seemingly full-length EVEs not associated with symptoms and smaller EVE pieces. The later are likely the result of EVE degradations or host defenses against invading nucleic acids during evolution, and might explain the first reports that *Ectocarpus* genome contains several viral DNA pieces (Delaroque and Boland, 2008).

Recent large-scale comparative genomic studies have revealed that viral integrations are a pervasive feature of brown algal genomes. Large fragments of giant double-stranded DNA viruses related to phaeoviruses have been detected across diverse brown algal lineages (Cock et al, 2010; Denoeud et al, 2024; Mckeown et al, 2025). A recent comprehensive analysis of dozens of newly sequenced genomes demonstrated that viral element insertions are not restricted to isolated taxa but are distributed throughout the brown algal phylogeny (Denoeud et al, 2024). This phylogenetic distribution suggests that brown alga-virus interactions have been recurrent over evolutionary timescales, leaving behind a substantial genomic signature. These genomic findings corroborate previous PCR-based surveys of natural populations that suggested widespread occurrence of viral sequences in algal communities (e.g., McKeown et al, 2018).

The viral sequences detected often comprise large portions of phaeoviral genomes, in some cases approaching near-complete genomes rather than short remnants. This observation aligns with the known biology of phaeoviruses, which integrate into the genomes of their hosts and can establish long-lasting, latent infections (Duchêne et al, 2025). While many of the integrated sequences identified are fragmented and degraded, their extent suggests that past infections involved major viral contributions to host genomes. These viral regions also include fragments with affinities to more distantly related nucleocytoviruses (*Imitervirales*) (Denoeud et al, 2024). These results reinforce the idea that phaeoviruses, and possibly other nucleocytoviruses, have not only shaped brown algal biology through infection cycles but have also contributed to the genomic architecture of their hosts and helped shaping their evolution.

From an evolutionary perspective, the widespread presence of viral genome fragments points to repeated episodes of endogenization during the diversification of brown algae. The diversity of phaeoviral EVEs and their non-congruence with brown algal phylogeny suggest repeated event of infection rather than a single event followed by duplication and diversification, but this remains to be further studied. Such events may have contributed to changes in brown algal genome size and structure, and potentially offered raw genetic material for functional innovation through the domestication of viral genes (Macaisne et al, 2017; Denoeud et al, 2024). In other words, although the activity and biological significance of most integrated elements remain to be determined, their ubiquity suggests that interactions with giant viruses have been a defining feature in the evolutionary history of brown algae (see “Ecological and evolutionary implications” section).

Together, these findings open new avenues for exploring how host–virus relationships extend beyond infection dynamics to leave lasting imprints on genome evolution. A striking example of this phenomenon comes from the filamentous brown alga *Porterinema*, whose genome was recently shown to contain multiple, diverse EVEs (Mckeown et al, 2025). The diversity and abundance of these viral insertions point to a history of repeated, independent integration events rather than a single viral endogenization followed by vertical transmission. In several cases, the integrated elements span extensive genomic regions, retaining gene repertoires typical of giant dsDNA viruses, including those involved in replication and virion structure. The *Porterinema* case study highlights the potential evolutionary and functional implications of viral endogenization. Some of the endogenous viral fragments appear relatively intact and may retain transcriptional activity, suggesting possible roles in host biology or viral reactivation. Others are highly degraded, consistent with ancient and non-functional remnants. Together, these findings demonstrate how repeated interactions with giant viruses have left a complex genomic legacy in *Porterinema*. Beyond documenting viral persistence, they emphasize the potential of endogenized elements to shape genome architecture, act as a source of genetic novelty, and provide a molecular fingerprint of long-term co-evolution between brown algae and their viruses.

### Mechanisms of integration, latency, and activation

Phaeoviruses exhibit an integration strategy that differentiates them from many other nucleocytoviruses. Unlike typical giant viruses such as mimiviruses, which mainly employ lytic replication strategies (Scola et al, 2003; Machado et al, 2022), phaeoviruses establish long-term latent infections by inserting their DNA into the nuclei of reproductive zoids in brown algae (Duchêne et al, 2025). This integration ensures vertical transmission of the viral genome through the germline, allowing the virus to persist for multiple generations (Müller, 1991; Duchêne et al, 2025). In contrast, other nucleocytoviruses generally leave minimal or no genomic traces in their hosts, as their life cycles rely on repeated horizontal transmission and lytic replication (Abergel and Claverie, 2020).

The only established Nucleocytoviricota model for which a latent life cycle is demonstrated are phaeoviruses (Duchêne et al, 2025), while in punuiviruses (Imitervirales, see Fig. 2A, Erazo-Garcia et al, 2025) a cryptic infection has been described. Some genomic evidence suggests that Xanthoviridae, infecting xanthophyte algae (from the proposed order ‘Pandoravirales’, Duchêne et al, 2025) and Mycodnaviridae, infecting fungi (Imitervirales, Myers et al, 2025) could also use a lysogenic life cycle.

Phaeoviruses are thought to integrate by site-specific recombination between a site in the algae genome, termed *att*B following phage nomenclature, and a site in the viral genome (termed *att*P), giving rise to the inserted virus flanked by *att*R and *att*L sites (Duchêne et al, 2025). The *att*P site is conserved among phaeoviruses, whereas the *att*B site contains only a short conserved CC motif, which is also present in *att*P (Fig. 2B). Note that the full diversity of phaeoviruses has not yet been explored. For example, viruses infecting the brown alga *Feldmannia* possss different *att*B/*att*P sequences, and integration occurs at CG sites (Meints et al, 2008). However, despite sequence divergence, all phaeoviruses including the *Feldmannia* viruses presumably integrate via a conserved mechanism. In both type of *att*P site, the insertion site (CC or CG) is flanked by inverted repeats that could lead to the formation of a hairpin exposing the CC/CG nucleotides. The integration is likely to occur via a tyrosine recombinase, which is conserved across phaeoviruses and contains the catalytic residues required for its function (Delaroque et al, 2001; Duchêne et al, 2025).

Xanthoviruses are a group of viruses that form a sister clade to phaeoviruses. To date, evidence for their existence comes exclusively from xanthophyte genomes carrying nucleocytoviruses hallmark viral genes, such as MCP and DNA polymerase B (Denoeud et al, 2024). Interestingly, these genomes also contain tyrosine recombinase genes that are phylogenetically sister to those of phaeoviruses, suggesting that xanthoviruses may employ the same enzymatic machinery to integrate into algal genomes and potentially share a similar latent life cycle strategy. Thus, although their sequences have diverged, the underlying integration mechanism is likely conserved across phaeoviruses and may also extend to xanthoviruses.

In contrast, punuiviruses, which may establish latent life cycles in the green algae *Chlamydomonas*, appear to utilize a completely different insertion mechanism (Erazo-Garcia et al, 2025). Integration of their genome results in target site duplications, a process that closely resembles transposon-mediated insertion (Fig. 2B). Their putative integrase is phylogenetically related to retroviral integrases and may be derived from virophages. Together, these comparisons highlight that while punuiviruses and phaeoviruses both belong to the *Nucleocytoviricota*, they may have evolved latent life cycles independently, relying on distinct enzymatic tools acquired from different evolutionary sources.

Environmental and developmental cues (e.g., temperature, onset of sexual reproduction) were suggested to break latency and activate phaeoviral EVEs. However, only recently has a clear link been established between activation of EVEs and the characteristic symptoms observed in infected *Ectocarpus*. Work by Duchêne et al provided this long-awaited proof, showing that reactivation of a specific EVE during reproductive development is directly associated with symptomatic phenotypes specifically in gametangia, which develop to produce viral particles instead of gametes (Duchêne et al, 2025). When transmitted to the descendance, either horizontally or vertically via the germline, these EVEs conserve their ability to reactivate. Interestingly, the phaeoviral genomes contains several sensory systems, in particular hybrid histidine kinases, that could play a role in the activation of the virus in response to developmental and environmental cues (Delaroque et al, 2001). In the *Chlamydomonas*-punuiviruses system, the exact trigger for viral activation remains unknown (Erazo-Garcia et al, 2025). The virus seems to activate as the culture ages, during mid-exponential to stationary phase of growth, suggesting that accumulation of metabolites or nutrient exhaustion could be triggers for viral activation. Temperature is a recurrent trigger for activation of eukaryotic viruses. For example, the mirusvirus which is found integrated in the protist *Aurantiochytrium limacinum* is transcribed after a cold shock experiment (Collier et al, 2023a; Chung et al, 2025). Another example is the green algae *Cylindrocapsa*, which produced viral particles in developing germling after a heat-shock treatment (Hoffman and Stanker, 1976). Finally, the established model for latent infection is temperate phages, which infect bacteria and remain latent in their genomes in the form of inserted prophages. Diverse environmental cues are known to trigger prophage induction, such as abiotic growth conditions (nutrients, (an)aerobic conditions, antibiotics, heavy metals, pollutants, ultraviolet radiation or temperature) but also biotic cues such as superinfection by other phages and host density (Bruce et al, 2021; Zhang et al, 2022). Whether environmental cues other than temperature are involved in phaeovirus activation remains to be investigated.

In the silent state, i.e., in the absence of viral symptoms, the inserted phaeoviruses are transcriptionally repressed, except for a handful of genes in that are constitutively expressed and could be involved in breaking the latency (Cock et al, 2010; Denoeud et al, 2024; Ban, 2025; Duchêne et al, 2025). New insights into the mechanisms involved in this repression are rising from the studies of *Ectocarpus* genome regulation. One method to study genome regulation is Hi-C, a genome-wide sequencing technique that captures the three-dimensional organization of chromatin by identifying physical interactions between different regions of DNA within the nucleus. Interestingly, Hi-C demonstrated that a silent EVE in *Ectocarpus* species 7 (strain Ec32) is sequestrated within higher-order 3D chromatin domains in close proximity to telomeres, likely preventing spurious activation under normal growth conditions (Liu et al, 2024). The same EVE region is enriched in repressive histone methylation marks H3K79 and depleted of activating chromatin modifications (Bourdareau et al, 2021; Gueno et al, 2022). Chromatin remodeling is involved in repression of *Nucleocytoviricota* EVEs in other systems. For example, the giant EVE in the fungi *Rhizophagus irregularis* is in a condensed chromatin region, insulated from the rest of the chromosome (Zhao et al, 2023). Although absent in brown algae (Vigneau et al, 2025), cytosine methylation is also recognized as a repressive mechanism to control foreign DNA. In the Discoba algae *Euglena*, cytosines within EVEs are heavily methylated, supposedly as part of the alga’s antiviral defense (Jivaji et al, 2025). This pattern extends beyond: in the protist *Amoebidium appalachense*, closely related to animals, 5-methylcytosine (5mC) silences giant virus insertions (Sarre et al, 2024). These findings suggest that 5mC DNA methylation, histone modification and other chromatin-mediated repression mechanisms represent conserved eukaryotic strategies for controlling newly acquired viral DNA, potentially enabling stable coexistence between host genomes and integrated viral elements.

Herpes viruses are a well-known example of latent viruses in eukaryotes, and herpesvirus relatives (mirusviruses) are also found in the genomes of some protists (Collier et al, 2023b). Herpes simplex virus (HSV) can establish lytic infection in epithelial cells or latency in sensory neurons, with chromatin state on viral DNA regulating this switch. Herpes Human Virus 6 (HHV-6A/B) genomes integrate into telomeres of latently infected cells. Following the establishment of latent infection, viral lytic gene expression is silenced, and the lytic gene promoters are associated with repressive heterochromatin (H3K9me2/3 and H3K27me3) (Knipe and Cliffe, 2008; Cliffe et al, 2009; Kwiatkowski et al, 2009; Cliffe et al, 2013; Nicoll et al, 2016; Suzich and Cliffe, 2018). These results indicate that viral genomes reside in condensed chromatin, revealing epigenetic mechanisms underlying integrated viral genome silencing and shared principles across organisms as phylogenetically distant as brown algae and humans.

While phaeovirus activation involves changes in temperature, virtually nothing is known about the molecular mechanisms underlying this process. No connection has yet been established between chromatin-based silencing in vegetative tissues and the release of silencing in specific cell types or in response to environmental cues. The virus has been shown to exist as a circular form in symptomatic algae (Delaroque et al, 2001; Duchêne et al, 2025), but the molecular basis of provirus reactivation remains largely unexplored. The genome of EsV-1 was found to contain proteins with similarities to bacteriophage regulators of lysogeny, but this will have to be functionally validated (Delaroque et al, 2001). It also remains to be explored whether giant viruses encode proteins that counteract host antiviral inactivation mechanisms, analogous to the silencing suppressors evolved by plant DNA viruses (Burgyán and Havelda, 2011; Pumplin and Voinnet, 2013; Zhao et al, 2016; Baulcombe, 2022; Zhang et al, 2025).

The establishment of a connection between chromatin-based silencing, EVEs reactivation, and disease symptoms, three decades after the initial discovery of EsV-1, marks a major advance in understanding how EVEs shape host development.

### Ecological and evolutionary implications

The direct impact of phaeovirus on brown algal fitness is difficult to measure. Symptoms appear only in reproductive stages and no phenotypic differences could be observed between infected and healthy algae at immature stages (del Campo et al, 1997). Infected algae might produce fewer offspring, as viral replication occurs in their reproductive structures and diverts resources from gamete or spore production, though this remains to be experimentally tested. Surprisingly, several studies suggest that more than half of algal natural populations, and in some cases up to 100% of individuals, carry viral markers in their genomes (Sengco et al, 1996; Dixon et al, 2000; Müller et al, 2000; McKeown et al, 2017; McKeown et al, 2018; Ruiz Martínez et al, 2023; Ban, 2025). This implies that the fitness cost of carrying an EVE may not be very high. However, considering that many EVEs are not active (see above, especially the examples from *Porterinema*, Mckeown et al, 2025), PCR-based detections can reflect relicts of past infections rather than active ones. In fact, only ~5% of *Ectocarpus* isolates from Sengco et al (1996) and Müller et al (2000) showed symptoms in culture. Similarly, Dixon et al studied the direct occurrence of symptoms in *Ectocarpus fasciculatus* populations, with only 5% of the sampled individuals showing obvious symptoms (Dixon et al, 2000). The low prevalence of symptoms from field samples led to the idea that the virus is primarily transmitted vertically rather than by de novo infections of uninfected gametes or spores by virus particles (Sengco et al, 1996; Müller et al, 2000). The balance between vertical and horizontal transmission in field populations and whether virus may manipulate or affect host life cycle in nature remain open questions. The use of genomic data of algal populations to investigate full EVE presence coupled with demographic and epidemiological modeling of the viral life cycle could help to elucidate the preferred mode of transmission (vertical vs horizontal) of phaeoviruses in different algal species, and to predict how the virus may affect algal life cycle.

As temperature seems to be a critical parameter in the virus life cycle, this can also influence the severity of symptoms and the impact on the algal fitness. In the context of global warming, algae populations, and in particular kelps, are challenged with increasing temperature and heat waves (Ling et al, 2009; Wernberg et al, 2010; Raybaud et al, 2013; Krumhansl et al, 2016; Arafeh-Dalmau et al, 2019; Suskiewicz et al, 2024). Whether viral infections act as an additional stressor or increased temperature alleviate the stress of viral infection could influence the resilience of kelp populations, but this remains not known.

The extent to which viral integration contributes to host speciation, population structure and intraspecific variation has not been thoroughly assessed. Different *Ectocarpus* species and strains carry different phaeoviral EVEs (Denoeud et al, 2024; Duchêne et al, 2025), as do other brown algae (McKeown et al, 2017; McKeown et al, 2018; Ruiz Martínez et al, 2023; Denoeud et al, 2024; Ban, 2025), but the extent to which this shaped evolutionary dynamics at the population and species scale is unknown. Similarly, in Chlorophyta, *Nucleocytoviricota* EVEs contribute to intraspecific variability in *Chlamydomonas reinhardtii* populations, and to the evolution of green algal genomes (Moniruzzaman et al, 2020; Moniruzzaman et al, 2022), but their role in driving host evolution is yet unclear.

Viral infection has shaped the evolution of multicellular organism by providing novel genetic material. One striking example is the evolution of the placenta in placental mammals, which represents a classic case of viral gene co-option, i.e., the recruitment of viral genes by the host (Mi et al, 2000; Chuong, 2018). This leads to the question: has co-option of viral genes significantly contributed to the evolution of multicellular development in brown algae? One example of potential viral gene co-option comes from the *Ectocarpus IMMEDIATE UPRIGHT (IMM)* gene, which plays a crucial role in cell type determination during early sporophyte development in *Ectocarpus* (Macaisne et al, 2017). The *IMM* gene encodes a protein containing repeated motifs also found in the *EsV-1-7* gene of the EsV-1. Brown algae possess large families of EsV-1-7 domain genes that are rare in other Stramenopiles, suggesting expansion of this family. Interestingly, some viral genes present in brown algal EVEs show signs of domestication such as heavy intronization or transcription without symptom production (i.e., from inactive EVEs), which highlights their potential to be co-opted by the algae (Denoeud et al, 2024; Mckeown et al, 2025). It will be necessary to examine whether these genes play a role in the development and evolution of their hosts, in order to clearly demonstrate the capacity for domesticated viral genes to transition from genomic ‘passengers’ to active contributors in host biology (Irwin et al, 2022).

The recent development of reverse genetic tools for brown algae (Badis et al, 2021; Martinho et al, 2025, see below) now enable to experimentally assess the roles of co-opted EVEs genes in host development and physiology. Interestingly, a substantial proportion of brown algal-specific genes are of viral origin (Barrera-Redondo et al, 2023; Denoeud et al, 2024), raising the intriguing possibility that viral integration and subsequent co-option have significantly contributed to the evolution of multicellular development in brown algae. This represents a potentially transformative mechanism whereby horizontal gene transfer from viruses has facilitated the acquisition of novel developmental capabilities, highlighting the evolutionary significance of host–virus interactions beyond traditional pathogenic contexts.

### Unlocking brown algal virology: the promise of new tools

Brown algae (Phaeophyceae), a highly diverse lineage of Stramenopiles that independently evolved complex multicellularity, exhibit a wide variety of morphologies, life cycles, and reproductive strategies, making them valuable model systems for investigating fundamental biological processes (Box 1). Over the past two decades, the brown alga *Ectocarpus* has moved from descriptive genomics into a phase where virology questions can be addressed mechanistically, as research groups worldwide have developed and adapted molecular and genetic tools (Coelho and Cock, 2020). Successive advances in genome assembly, from the first *Ectocarpus* reference genome (Cock et al, 2010) to highly contiguous chromosome-scale and near telomere-to-telomere genomes (Cormier et al, 2017; Liu et al, 2024; Barrera-Redondo et al, 2025), now allow to identify EVEs precise genomic location. These resources are complemented by transcriptomic atlases that chart the developmental program of *Ectocarpus* (Lipinska et al, 2015; Luthringer et al, 2015; Cossard et al, 2022; Lotharukpong et al, 2024; Ratchinski et al, 2025), that can be used to study expression of EVE genes across life-cycle stages. Together these tools provide a solid foundation for functional studies and a robust baseline against which viral influence can be detected and quantified.

Functional genetic tools are increasingly accessible for the brown algae. Forward genetic screens using UV or EMS mutagenesis coupled to mapping have identified regulators of development and life-cycle transitions, yielding clear genotype–phenotype links (e.g., Coelho et al, 2011; Godfroy et al, 2017; Macaisne et al, 2017; Arun et al, 2019). Reverse genetics tools have advanced rapidly. Reverse genetics initially leveraged RNA interference for targeted knockdowns, but the introduction of CRISPR-Cas9 has been a major breakthrough (Farnham et al, 2013; Badis et al, 2021; Martinho et al, 2025). Optimized delivery by biolistic bombardment and laser-assisted microinjection introduces DNA or CRISPR-Cas9 RNPs into reproductive cells to enable precise knockouts and targeted mutagenesis. Selection using APT/2-FA exploit the haploid phase of brown algae, making recovery and phenotyping of edited lines efficient without need for backcrossing. Improved PEG-mediated RNP delivery system with CRISPR-Cas12-based genome editing method (Martinho et al, 2025) and the development of knock-in strategies should extend precise editing beyond *Ectocarpus* to additional taxa. These tools have already revealed key regulators of sex determination and sporophyte development (Luthringer et al, 2024; Martinho et al, 2025), and lay the groundwork for reporter tagging and locus-specific manipulations in *Ectocarpus* and other brown algal lineages.

Advances in transcriptomics and epigenomics have yielded temporal and regulatory insights into *Ectocarpus* development (Lipinska et al, 2015; Lotharukpong et al, 2024; Vigneau et al, 2024; Ratchinski et al, 2025). These datasets provide a quantitative framework against which viral perturbations can be detected and interpreted, supporting future efforts to identify potential viral imprints on developmental pathways and defense responses. To address the molecular mechanisms underlying the host–virus interaction, a viable possibility is low‑input RNA‑seq on microdissected, virus‑affected reproductive tissues (e.g., gametangia/sporangia), enabling library construction from minute samples and tissue‑level dissection of host–virus interactions in compartments where replication is concentrated. In parallel, single‑cell and single‑nucleus RNA‑seq, already widely used in other host–virus systems, when adapted to brown algae will resolve cell‑type‑specific infection states and trajectories of EVE reactivation. Experience from mammalian and plant infections points to marked cell‑to‑cell heterogeneity, with reservoir‑like cells coexisting alongside exposed yet uninfected cells, and distinct cell identities orchestrating spread or persistence (Steuerman et al, 2018; Liao et al, 2020; Tang et al, 2023; Zhu et al, 2023). In brown algae, such approaches should pinpoint when and where EVEs reactivate and which cell types permit productive replication; beyond activation states, single‑cell and single‑nucleus RNA‑seq can delineate the transcriptional circuits, signaling modules, and cell–cell communication that mediate host–virus interplay at cellular resolution. Moreover, ChIP‑seq and Hi‑C offer complementary views of EVE silencing, repressive histone marks and three‑dimensional chromatin landscapes (Bourdareau et al, 2021; Liu et al, 2024).

Imaging approaches are increasingly being used to localize infection in space and time (e.g., Mayer et al, 2025). Coupled with confocal microscopy, RNA fluorescence in *situ* hybridization visualizes viral or host transcripts within virus‑active tissues, while immunofluorescence allows to map host pathways during antiviral responses and the subcellular distribution of viral proteins (Vincent et al, 2021; Mayer et al, 2025). Together, these approaches render the spatiotemporal dynamics of infection tractable. Electron microscopy provides ultrastructural views of virion assembly sites and infection foci (Maier et al, 2002; Aicher et al, 2021; Rigou et al, 2025), and expansion microscopy, has the potential to yield subcellular resolution in algal tissues (Gambarotto et al, 2019; Klena et al, 2023). Applied in concert, these imaging tools are expected to reveal organelle remodeling and membrane/cytoskeletal reorganization that accompany virus activation in the algal reproductive structures.

Taken together, these technical developments are expected to revolutionize brown algal virology. The final section below outlines future directions and open questions that these tools now bring within reach.

## Future directions and open questions

The expanding genetic toolkit in brown algae offers an unprecedented opportunity to dissect virus–host interactions in this lineage. Delivery methods such as biolistics, PEG-mediated transformation, and microinjection can be used to introduce viral DNA, reporter constructs, or engineered host genes directly into algal cells, enabling controlled infection assays and functional studies (Jiang et al, 2003; Zhang et al, 2008; Shen et al, 2023; Martinho et al, 2025). Forward genetic approaches, including UV or EMS mutagenesis coupled to mapping, can generate host variants with altered susceptibility or resistance to viral activation, revealing the genetic determinants of infection outcomes. The haploid life stage of *Ectocarpus* greatly facilitates such screens, although careful experimental design is required because fertility-dependent phenotypes may be delayed. Natural variation can likewise be leveraged to identify loci underlying differential susceptibility, paralleling strategies in model plants.

Reverse genetics approaches, including RNA interference and CRISPR/Cas-mediated genome editing, permit targeted knockdowns or knockouts of host genes involved in viral silencing, replication, or reactivation, and can be extended to interrogate specific viral gene functions (Farnham et al, 2013; Godfroy et al, 2017; Macaisne et al, 2017; Badis et al, 2021; Shen et al, 2023; Luthringer et al, 2024; Martinho et al, 2025). Advances toward precise knock-ins and reporter tagging on viral or host alleles, would allow direct visualization of virus–host interactions in vivo and, together with base/prime editing, enable fine-scale test of causal pathways.

Several questions now come within reach. Genomic and chromatin analyses should clarify mechanisms of integration and the nuclear positioning and chromatin states that enforce latency, and whether developmental or environmental cues remodel these states to permit activation. At the cellular level, tagged viral or host components can allow to visualize infection dynamics in real time, while natural variation and forward genetics could reveal loci controlling resistance or susceptibility. Extending these approaches beyond *Ectocarpus* to other brown algal species promises to uncover lineage-specific virus–host strategies and provide a broader understanding of viral biology in multicellular eukaryotes. Collectively, the expanding toolkit of forward and reverse genetics positions brown algae as a highly tractable system for exploring fundamental principles of virus–host interactions and the evolution of latent viral strategies. In parallel, multi-omics approaches such as hybrid genome/virome sequencing, RNA-seq at bulk and single-cell/-nucleus resolution, epigenomics, proteomics, metabolomics, and spatial imaging, provide the integrative readouts that connect these mechanisms to activation and transmission in natural populations. Together, these advances mark an exciting era for research on giant viruses and brown algae, offering unprecedented opportunities to unravel the complexities of virus–host interactions across diverse ecological and evolutionary contexts.

## Supplementary information

Peer Review File

## Glossary

**Chronic infection**: persistent infection in which viral particles are continuously produced
**EVE** (Endogenous Viral Element): genetic element in a host genome derived from a virus sequence. EVEs can represent full-length viruses or fragments of viral genomes, and can derive from active integration mechanisms or accidental endogenization of a replicating virus.
**Gametophyte**: One of the two generations (usually haploid and multicellular) in plants and algae that undergo alternation of generations. Here, gametophytes develop from spores and give rise to the male and female gametes.
**Latent infection**: persistent infections in which complete copies of the viral genome persist in the host for extended periods without the continuous production of infectious virus.
**Lytic infection**: active replication of the viral genome, characterized by the release of new progeny virus particles, often upon the lysis of the host cell.
**Sporophyte**: One of the two generations (usually diploid and multicellular) in the life cycle of plants and algae that undergo alternation of generations. Here, the sporophyte produces haploid spores through meiosis that develop into a gametophyte.

## References

[CR1] Abergel C, Claverie J-M (2020) Giant viruses. Curr Biol 30:R1108–R1110 33022247 10.1016/j.cub.2020.08.055

[CR2] Aicher S-M, Monaghan P, Netherton CL, Hawes PC (2021) Unpicking the secrets of African swine fever viral replication sites. Viruses 13:77 33429879 10.3390/v13010077 PMC7827680

[CR3] Arafeh-Dalmau N, Montaño-Moctezuma G, Martínez JA, Beas-Luna R, Schoeman DS, Torres-Moye G (2019) Extreme marine heatwaves alter kelp forest community near its equatorward distribution limit. Front Mar Sci. 10.3389/fmars.2019.00499

[CR4] Arun A, Coelho SM, Peters AF, Bourdareau S, Pérès L, Scornet D, Strittmatter M, Lipinska AP, Yao H, Godfroy O et al (2019) Convergent recruitment of TALE homeodomain life cycle regulators to direct sporophyte development in land plants and brown algae. eLife 8:e43101 30644818 10.7554/eLife.43101 PMC6368402

[CR5] Aylward FO, Abrahão JS, Brussaard CPD, Fischer MG, Moniruzzaman M, Ogata H, Suttle CA (2023) Taxonomic update for giant viruses in the order Imitervirales (phylum Nucleocytoviricota). Arch Virol 168:283 37904060 10.1007/s00705-023-05906-3 PMC11230039

[CR6] Aylward FO, Moniruzzaman M, Ha AD, Koonin EV (2021) A phylogenomic framework for charting the diversity and evolution of giant viruses. PLOS Biol 19:e3001430 34705818 10.1371/journal.pbio.3001430 PMC8575486

[CR7] Badis Y, Scornet D, Harada M, Caillard C, Godfroy O, Raphalen M, Gachon CMM, Coelho SM, Motomura T, Nagasato C et al (2021) Targeted CRISPR-Cas9-based gene knockouts in the model brown alga *Ectocarpus*. New Phytol 231:2077–2091 10.1111/nph.17525 34076889

[CR8] Ban H (2025) Infection signatures of multiple Nucleocytoviricota virus lineages in the brown algae Undaria pinnatifida revealed by population-wide genome analysis. J Appl Phycol 37:2705–2714

[CR9] Barrera-Redondo J, Lipinska AP, Liu P, Dinatale E, Cossard G, Bogaert K, Hoshino M, Craig RJ, Avia K, Leiria G, et al (2025) Origin and evolutionary trajectories of brown algal sex chromosomes. Nat Ecol Evol 9:2127–2144 10.1038/s41559-025-02838-w PMC12592211 40855228

[CR10] Barrera-Redondo J, Lotharukpong JS, Drost H-G, Coelho SM (2023) Uncovering gene-family founder events during major evolutionary transitions in animals, plants and fungi using GenEra. Genome Biol 24:54 36964572 10.1186/s13059-023-02895-z PMC10037820

[CR11] Batista RA, Wang L, Bogaert KA, Coelho SM (2024) Insights into the molecular bases of multicellular development from brown algae. Development 151:dev203004 39302848 10.1242/dev.203004

[CR12] Baulcombe DC (2022) The role of viruses in identifying and analyzing RNA silencing. Annu Rev Virol 9:353–373 35655339 10.1146/annurev-virology-091919-064218

[CR13] Bignaud A, Conti DE, Thierry A, Serizay J, Labadie K, Poulain J, Cheny O, Colón-González M, Debarbieux L, Guerrero-Osornio M, et al (2025) Phages with a broad host range are common across ecosystems. Nat Microbiol 10:2537–2549 10.1038/s41564-025-02108-2 40973790

[CR14] Bourdareau S, Tirichine L, Lombard B, Loew D, Scornet D, Wu Y, Coelho SM, Cock JM (2021) Histone modifications during the life cycle of the brown alga Ectocarpus. Genome Biol 22:12 33397407 10.1186/s13059-020-02216-8 PMC7784034

[CR15] Bruce JB, Lion S, Buckling A, Westra ER, Gandon S (2021) Regulation of prophage induction and lysogenization by phage communication systems. Curr Biol CB 31:5046–5051.e7 34562385 10.1016/j.cub.2021.08.073 PMC8612742

[CR16] Burgyán J, Havelda Z (2011) Viral suppressors of RNA silencing. Trends Plant Sci 16:265–272 21439890 10.1016/j.tplants.2011.02.010

[CR17] Byl P, Schvarcz CR, Thomy J, Li Q, Williams CB, LaButti K, Schulz F, Edwards KF, Steward GF (2025) Evidence for the acquisition of a proteorhodopsin-like rhodopsin by a chrysophyte-infecting giant virus. Preprint at 10.1101/2025.06.17.660233 10.1128/jvi.00520-26 PMC13595923 42573254

[CR18] Cho A, Tikhonenkov DV, Lax G, Prokina KI, Keeling PJ (2024) Phylogenomic position of genetically diverse phagotrophic stramenopile flagellates in the sediment-associated MAST-6 lineage and a potentially halotolerant placididean. Mol Phylogenet Evol 190:107964 37951557 10.1016/j.ympev.2023.107964

[CR19] Chung D, Brask N, Matar S, Gallot-Lavallée L, Pringle E, Duguay B, Blais C, Latimer J, Slamovits C, Leyland B et al (2025) Persistent mirusvirus infection in the marine protist Aurantiochytrium. Nat Commun 16:9922. 10.21203/rs.3.rs-5686297/v1 10.1038/s41467-025-65172-6 PMC12606086 41219190

[CR20] Chuong EB (2018) The placenta goes viral: Retroviruses control gene expression in pregnancy. PLoS Biol 16:e3000028 30300353 10.1371/journal.pbio.3000028 PMC6177113

[CR21] Cliffe AR, Coen DM, Knipe DM (2013) Kinetics of facultative heterochromatin and polycomb group protein association with the herpes simplex viral genome during establishment of latent infection. mBio 4: 10.1128/mbio.00590-12 10.1128/mBio.00590-12 PMC3551550 23322639

[CR22] Cliffe AR, Garber DA, Knipe DM (2009) Transcription of the herpes simplex virus latency-associated transcript promotes the formation of facultative heterochromatin on lytic promoters. J Virol 83:8182–8190 19515781 10.1128/JVI.00712-09 PMC2715743

[CR23] Clitheroe SB, Evans LV (1974) Viruslike particles in the brown alga Ectocarpus. J Ultrastruct Res 49:211–217 4431075 10.1016/s0022-5320(74)80032-8

[CR24] Cock JM, Sterck L, Rouzé P, Scornet D, Allen AE, Amoutzias G, Anthouard V, Artiguenave F, Aury J-M, Badger JH et al (2010) The Ectocarpus genome and the independent evolution of multicellularity in brown algae. Nature 465:617–621 20520714 10.1038/nature09016

[CR25] Coelho SM, Cock JM (2020) Brown algal model organisms. Annu Rev Genet 54:71–92 33228413 10.1146/annurev-genet-030620-093031

[CR26] Coelho SM, Godfroy O, Arun A, Le Corguillé G, Peters AF, Cock JM (2011) OUROBOROS is a master regulator of the gametophyte to sporophyte life cycle transition in the brown alga Ectocarpus. Proc Natl Acad Sci USA 108:11518–11523 21709217 10.1073/pnas.1102274108 PMC3136289

[CR27] Coelho SM, Taylor AR, Ryan KP, Sousa-Pinto I, Brown MT, Brownlee C (2002) Spatiotemporal patterning of reactive oxygen production and Ca2+ wave propagation in fucus rhizoid cells. Plant Cell 14:2369–2381 12368492 10.1105/tpc.003285 PMC151223

[CR28] Collier JL, Rest JS, Gallot-Lavallée L, Lavington E, Kuo A, Jenkins J, Plott C, Pangilinan J, Daum C, Grigoriev IV et al (2023a) The protist Aurantiochytrium has universal subtelomeric rDNAs and is a host for mirusviruses. Curr Biol 33:5199–5207.e4 37913769 10.1016/j.cub.2023.10.009

[CR29] Collier JL, Rest JS, Gallot-Lavallée L, Lavington E, Kuo A, Jenkins J, Plott C, Pangilinan J, Daum C, Grigoriev IV et al (2023b) The protist Aurantiochytrium has universal subtelomeric rDNAs and is a host for mirusviruses. Curr Biol 33:5199–5207.e4 37913769 10.1016/j.cub.2023.10.009

[CR30] Cormier A, Avia K, Sterck L, Derrien T, Wucher V, Andres G, Monsoor M, Godfroy O, Lipinska A, Perrineau M et al (2017) Re-annotation, improved large-scale assembly and establishment of a catalogue of noncoding loci for the genome of the model brown alga *Ectocarpus*. New Phytol 214:219–232 27870061 10.1111/nph.14321

[CR31] Cossard GG, Godfroy O, Nehr Z, Cruaud C, Cock JM, Lipinska AP, Coelho SM (2022) Selection drives convergent gene expression changes during transitions to co-sexuality in haploid sexual systems. Nat Ecol Evol 6:579–589 35314785 10.1038/s41559-022-01692-4 PMC9085613

[CR32] del Campo E, Ramazanov Z, Garcia-Reina G, Müller DG (1997) Photosynthetic responses and growth performance of virus-infected and noninfected Ectocarpus siliculosus (Phaeophyceae). Phycologia 36:186–189

[CR33] Delaroque N, Boland W (2008) The genome of the brown alga Ectocarpus siliculosus contains a series of viral DNA pieces, suggesting an ancient association with large dsDNA viruses. BMC Evol Biol 8:110 18405387 10.1186/1471-2148-8-110 PMC2373305

[CR34] Delaroque N, Maier I, Knippers R, Müller DG (1999) Persistent virus integration into the genome of its algal host, Ectocarpus siliculosus (Phaeophyceae). J Gen Virol 80:1367–1370 10374952 10.1099/0022-1317-80-6-1367

[CR35] Delaroque N, Müller DG, Bothe G, Pohl T, Knippers R, Boland W (2001) The complete DNA sequence of the Ectocarpus siliculosus virus EsV-1 genome. Virology 287:112–132 11504547 10.1006/viro.2001.1028

[CR36] Delaroque N, Wolf S, Müller DG, Knippers R (2000a) The brown algal virus EsV-1 particle contains a putative hybrid histidine kinase. Virology 273:383–390 10915609 10.1006/viro.2000.0383

[CR37] Delaroque N, Wolf S, Müller DG, Knippers R (2000b) Characterization and immunolocalization of major structural proteins in the brown algal virus EsV-1. Virology 269:148–155 10725207 10.1006/viro.2000.0225

[CR38] Denoeud F, Godfroy O, Cruaud C, Heesch S, Nehr Z, Tadrent N, Couloux A, Brillet-Guéguen L, Delage L, Mckeown D et al (2024) Evolutionary genomics of the emergence of brown algae as key components of coastal ecosystems. Cell 187:6943–6965.e39 39571576 10.1016/j.cell.2024.10.049

[CR39] Dixon NM, Leadbeater BSC, Wood KR (2000) Frequency of viral infection in a field population of Ectocarpus fasciculatus (Ectocarpales, Phaeophyceae). Phycologia 39:258–263

[CR40] Duchêne C, Craig RJ, Martinho C, Luthringer R, Agullo F, Hipp K, Escudeiro P, Alva V, Haas FB, Coelho SM (2025) Latent endogenous viral elements drive active infection and inheritance in a multicellular host. Preprint at 10.1101/2025.09.15.676377 10.1038/s41564-026-02361-z PMC13236599 42129496

[CR41] Endo H, Blanc-Mathieu R, Li Y, Salazar G, Henry N, Labadie K, de Vargas C, Sullivan MB, Bowler C, Wincker P et al (2020) Biogeography of marine giant viruses reveals their interplay with eukaryotes and ecological functions. Nat Ecol Evol 4:1639–1649 32895519 10.1038/s41559-020-01288-w

[CR42] Erazo-Garcia MP, Sheyn U, Barth ZK, Craig RJ, Wessman P, Jivaji AM, Ray WK, Svensson-Coelho M, Cornwallis CK, Rengefors K, et al (2025) Cryptic infection of a giant virus in a unicellular green alga. Science 388:eads6303 10.1126/science.ads6303 PMC12147526 40208960

[CR43] Farnham G, Strittmatter M, Coelho S, Cock JM, Brownlee C (2013) Gene silencing in Fucus embryos: developmental consequences of RNAi-mediated cytoskeletal disruption. J Phycol 49:819–829 27007308 10.1111/jpy.12096

[CR44] Fischer MG (2016) Giant viruses come of age. Curr Opin Microbiol 31:50–57 26999382 10.1016/j.mib.2016.03.001

[CR45] Forterre P, Gaïa M (2016) Giant viruses and the origin of modern eukaryotes. Curr Opin Microbiol 31:44–49 26894379 10.1016/j.mib.2016.02.001

[CR46] Fromm A, Hevroni G, Vincent F, Schatz D, Martinez-Gutierrez CA, Aylward FO, Vardi A (2024) Single-cell RNA-seq of the rare virosphere reveals the native hosts of giant viruses in the marine environment. Nat Microbiol 9:1619–1629 10.1038/s41564-024-01669-y PMC11265207 38605173

[CR47] Gallot-Lavallée L, Blanc G (2017) A glimpse of nucleo-cytoplasmic large DNA virus biodiversity through the eukaryotic genomics window. Viruses 9:17 28117696 10.3390/v9010017 PMC5294986

[CR48] Gambarotto D, Zwettler FU, Le Guennec M, Schmidt-Cernohorska M, Fortun D, Borgers S, Heine J, Schloetel J-G, Reuss M, Unser M et al (2019) Imaging cellular ultrastructures using expansion microscopy (U-ExM). Nat Methods 16:71–74 30559430 10.1038/s41592-018-0238-1 PMC6314451

[CR49] Godfroy O, Uji T, Nagasato C, Lipinska AP, Scornet D, Peters AF, Avia K, Colin S, Mignerot L, Motomura T et al (2017) DISTAG/TBCCd1 is required for basal cell fate determination in *Ectocarpus*. Plant Cell 29:3102–3122 29208703 10.1105/tpc.17.00440 PMC5757272

[CR50] Gueno J, Borg M, Bourdareau S, Cossard G, Godfroy O, Lipinska A, Tirichine L, Cock JM, Coelho SM (2022) Chromatin landscape associated with sexual differentiation in a UV sex determination system. Nucleic Acids Res 50:3307–3322 35253891 10.1093/nar/gkac145 PMC8989524

[CR51] Hevroni G, Vincent F, Ku C, Sheyn U, Vardi A (2023) Daily turnover of active giant virus infection during algal blooms revealed by single-cell transcriptomics. Sci Adv 9:eadf7971 37824628 10.1126/sciadv.adf7971 PMC10569711

[CR52] Hoffman LR, Stanker LH (1976) Virus-like particles in the green alga *Cylindrocapsa*. Can J Bot 54:2827–2841

[CR53] Irwin NAT, Pittis AA, Richards TA, Keeling PJ (2022) Systematic evaluation of horizontal gene transfer between eukaryotes and viruses. Nat Microbiol 7:327–336 34972821 10.1038/s41564-021-01026-3

[CR54] Jiang P, Qin S, Tseng CK (2003) Expression of the lacZ reporter gene in sporophytes of the seaweed Laminaria japonica (Phaeophyceae) by gametophyte-targeted transformation. Plant Cell Rep 21:1211–1216 12819923 10.1007/s00299-003-0645-2

[CR55] Jivaji AM, Karki S, Franken E, Erazo-Garcia MP, Barth ZK, Aylward FO (2025) Giant endogenous viral elements in the genome of the model protist Euglena gracilis reveal past interactions with giant viruses. J Virol 0:e00713-25 10.1128/jvi.00713-25 PMC12455990 40856505

[CR56] Joffe N, Kuhlisch C, Schleyer G, Ahlers NS, Shemi A, Vardi A (2024) Cell-to-cell heterogeneity drives host–virus coexistence in a bloom-forming alga. ISME J 18:wrae038 38452203 10.1093/ismejo/wrae038 PMC10980834

[CR57] Johannessen TV, Bratbak G, Larsen A, Ogata H, Egge ES, Edvardsen B, Eikrem W, Sandaa R-A (2015) Characterisation of three novel giant viruses reveals huge diversity among viruses infecting Prymnesiales (Haptophyta). Virology 476:180–188 25546253 10.1016/j.virol.2014.12.014

[CR58] Kapp M, Knippers R, Muller DG (1997) New members of a group of DNA viruses infecting brown algae. Phycol Res 45:85–90

[CR59] Klein M, Lanka STJ, Knippers R, Moller DG (1995) Coat protein of the Ectocarpus siliculosus virus. Virology 206:520–526 7831806 10.1016/s0042-6822(95)80068-9

[CR60] Klena N, Maltinti G, Batman U, Pigino G, Guichard P, Hamel V (2023) An in-depth guide to the ultrastructural expansion microscopy (U-ExM) of Chlamydomonas reinhardtii. Bio-Protoc. 10.21769/BioProtoc.4792 10.21769/BioProtoc.4792 PMC10502176 37719077

[CR61] Knipe DM, Cliffe A (2008) Chromatin control of herpes simplex virus lytic and latent infection. Nat Rev Microbiol 6:211–221 18264117 10.1038/nrmicro1794

[CR62] Koonin EV, Kuhn JH, Dolja VV, Krupovic M (2024) Megataxonomy and global ecology of the virosphere. ISME J 18:wrad042 38365236 10.1093/ismejo/wrad042 PMC10848233

[CR63] Krumhansl KA, Okamoto DK, Rassweiler A, Novak M, Bolton JJ, Cavanaugh KC, Connell SD, Johnson CR, Konar B, Ling SD et al (2016) Global patterns of kelp forest change over the past half-century. Proc Natl Acad Sci USA 113:13785–13790 27849580 10.1073/pnas.1606102113 PMC5137772

[CR64] Ku C, Sheyn U, Sebé-Pedrós A, Ben-Dor S, Schatz D, Tanay A, Rosenwasser S, Vardi A (2020) A single-cell view on alga-virus interactions reveals sequential transcriptional programs and infection states. Sci Adv 6:eaba4137 32490206 10.1126/sciadv.aba4137 PMC7239649

[CR65] Kuhlisch C, Schleyer G, Shahaf N, Vincent F, Schatz D, Vardi A (2021) Viral infection of algal blooms leaves a unique metabolic footprint on the dissolved organic matter in the ocean. Sci Adv 7:eabf4680 34144983 10.1126/sciadv.abf4680 PMC8213229

[CR66] Kwiatkowski DL, Thompson HW, Bloom DC (2009) The polycomb group protein Bmi1 binds to the herpes simplex virus 1 latent genome and maintains repressive histone marks during latency. J Virol 83:8173–8181 19515780 10.1128/JVI.00686-09 PMC2715759

[CR67] Lanka STJ, Klein M, Ramsperger U, Müller DG, Knippers R (1993) Genome structure of a virus infecting the marine brown alga Ectocarpus siliculosus. Virology 193:802–811 8460486 10.1006/viro.1993.1189

[CR68] Lee AM, Ivey RG, Henry EC, Meints RH (1995) Characterization of a repetitive DNA element in a brown algal virus. Virology 212:474–480 7571417 10.1006/viro.1995.1505

[CR69] Liao M, Liu Y, Yuan J, Wen Y, Xu G, Zhao J, Cheng L, Li J, Wang X, Wang F et al (2020) Single-cell landscape of bronchoalveolar immune cells in patients with COVID-19. Nat Med 26:842–844 32398875 10.1038/s41591-020-0901-9

[CR70] Ling SD, Johnson CR, Frusher SD, Ridgway KR (2009) Overfishing reduces resilience of kelp beds to climate-driven catastrophic phase shift. Proc Natl Acad Sci USA 106:22341–22345 20018706 10.1073/pnas.0907529106 PMC2793314

[CR71] Lipinska A, Cormier A, Luthringer R, Peters AF, Corre E, Gachon CMM, Cock JM, Coelho SM (2015) Sexual dimorphism and the evolution of sex-biased gene expression in the brown alga Ectocarpus. Mol Biol Evol 32:1581–1597 25725430 10.1093/molbev/msv049

[CR72] Liu P, Vigneau J, Craig RJ, Barrera-Redondo J, Avdievich E, Martinho C, Borg M, Haas FB, Liu C, Coelho SM (2024) 3D chromatin maps of a brown alga reveal U/V sex chromosome spatial organization. Nat Commun 15:9590 10.1038/s41467-024-53453-5 PMC11541908 39505852

[CR73] Luthringer R, Lipinska AP, Roze D, Cormier A, Macaisne N, Peters AF, Cock JM, Coelho SM (2015) The pseudoautosomal regions of the U/V sex chromosomes of the brown alga Ectocarpus exhibit unusual features. Mol Biol Evol 32:2973–2985 26248564 10.1093/molbev/msv173 PMC4610043

[CR74] Lotharukpong JS, Zheng M, Luthringer R, Liesner D, Drost H-G, Coelho SM (2024) A transcriptomic hourglass in brown algae. Nature 635:129–135 10.1038/s41586-024-08059-8 PMC11540847 39443791

[CR75] Luthringer R, Raphalen M, Guerra C, Colin S, Martinho C, Zheng M, Hoshino M, Badis Y, Lipinska AP, Haas FB et al (2024) Repeated co-option of HMG-box genes for sex determination in brown algae and animals. Science 383:eadk5466 38513029 10.1126/science.adk5466

[CR76] Macaisne N, Liu F, Scornet D, Peters AF, Lipinska A, Perrineau M-M, Henry A, Strittmatter M, Coelho SM, Cock JM (2017) The Ectocarpus IMMEDIATE UPRIGHT gene encodes a member of a novel family of cysteine-rich proteins with an unusual distribution across the eukaryotes. Development 144:409–418 28049657 10.1242/dev.141523

[CR77] Machado TB, de Aquino ILM, Abrahão JS (2022) Isolation of giant viruses of Acanthamoeba castellanii. Curr Protoc 2:e455 35612516 10.1002/cpz1.455

[CR78] Maier I, Müller DG, Katsaros C (2002) Entry of the DNA virus, Ectocarpus fasciculatus virus type 1 (Phycodnaviridae), into host cell cytosol and nucleus. Phycol Res 50:227–231

[CR79] Martinho C, Hoshino M, Raphalen M, Bukhanets V, Kerur A, Bogaert K, Luthringer R, Coelho S (2025) Efficient CRISPR–Cas genome editing in brown algae. Preprint at 10.1101/2025.07.21.665871 10.1016/j.crmeth.2025.101273 PMC12853178 41475352

[CR80] Mayer L, Nikolov G, Kunert M, Horn M, Willemsen A (2025) Mimivirus transcription and translation occur at well-defined locations within amoeba host cells. J Virol 99:e0055425 40511921 10.1128/jvi.00554-25 PMC12282054

[CR81] Mckeown D, Cormier A, Schroeder D, Couloux A, Tadrent N, Cock JM, Corre E (2025) Multiple, diverse endogenous giant virus elements within the genome of a brown alga. Virus Evol 11:veaf009 40061209 10.1093/ve/veaf009 PMC11890097

[CR82] McKeown D, Schroeder J, Stevens K, Peters A, Sáez C, Park J, Rothman M, Bolton J, Brown M, Schroeder D (2018) Phaeoviral infections are present in Macrocystis, Ecklonia and Undaria (Laminariales) and are influenced by wave exposure in Ectocarpales. Viruses 10:410 30081590 10.3390/v10080410 PMC6116031

[CR83] McKeown DA, Stevens K, Peters AF, Bond P, Harper GM, Brownlee C, Brown MT, Schroeder DC (2017) Phaeoviruses discovered in kelp (Laminariales). ISME J 11:2869–2873 28742072 10.1038/ismej.2017.130 PMC5702736

[CR84] Meints RH, Ivey RG, Lee AM, Choi T-J (2008) Identification of two virus integration sites in the brown alga feldmannia chromosome. J Virol 82:1407–1413 18032486 10.1128/JVI.01983-07 PMC2224422

[CR85] Mi S, Lee X, Li X, Veldman GM, Finnerty H, Racie L, LaVallie E, Tang X-Y, Edouard P, Howes S et al (2000) Syncytin is a captive retroviral envelope protein involved in human placental morphogenesis. Nature 403:785–789 10693809 10.1038/35001608

[CR86] Mojica KDA, Brussaard CPD (2025) Marine viruses and their role in marine ecosystems and carbon cycling. Annu Rev Mar Sci 18. 10.1146/annurev-marine-040324-020244 10.1146/annurev-marine-040324-020244 40780963

[CR87] Moniruzzaman M, Erazo-Garcia MP, Aylward FO (2022) Endogenous giant viruses contribute to intraspecies genomic variability in the model green alga *Chlamydomonas reinhardtii*. Virus Evol 8:veac102 36447475 10.1093/ve/veac102 PMC9693826

[CR88] Moniruzzaman M, Weinheimer AR, Martinez-Gutierrez CA, Aylward FO (2020) Widespread endogenization of giant viruses shapes genomes of green algae. Nature 588:141–145 33208937 10.1038/s41586-020-2924-2

[CR89] Müller DG (1991) Mendelian segregation of a virus genome during host meiosis in the marine brown alga Ectocarpus siliculosus. J Plant Physiol 137:739–743

[CR90] Müller DG, Kapp M, Knippers R (1998) Viruses in marine brown algae. Adv Virus Res 50:49–67 10.1016/s0065-3527(08)60805-2 9520996

[CR91] Müller DG, Kawai H, Stache B, Lanka S (1990) A virus infection in the marine brown alga *Ectocarpus siliculosus* (Phaeophyceae). Bot Acta 103:72–82

[CR92] Müller DG, Stache B (1992) Worldwide occurrence of virus-infections in filamentous marine brown algae. Helgoländer Meeresunters 46:1–8

[CR93] Müller DG, Westermeier R, Morales J, Reina GG, Campo E, del, Correa JA, Rometscha E (2000) Massive prevalence of viral DNA in Ectocarpus (Phaeophyceae, Ectocarpales) from two habitats in the North Atlantic and South Pacific. Botanica Marina 43:157–159

[CR94] Myers JM, Schulz F, Rahimlou S, Yadav V, Amses KR, Simmons DR, Sun S, Orozco-Quime M, Heitman J, Stajich JE et al (2025) Discovery of giant viruses as past and present infections of zoosporic fungi. Preprint at 10.1101/2024.01.04.574182

[CR95] Nagasaki K, Tomaru Y, Tarutani K, Katanozaka N, Yamanaka S, Tanabe H, Yamaguchi M (2003) Growth Characteristics and intraspecies host specificity of a large virus infecting the dinoflagellate Heterocapsa circularisquama. Appl Environ Microbiol 69:2580–2586 12732524 10.1128/AEM.69.5.2580-2586.2003 PMC154496

[CR96] Needham DM, Yoshizawa S, Hosaka T, Poirier C, Choi CJ, Hehenberger E, Irwin NAT, Wilken S, Yung C-M, Bachy C et al (2019) A distinct lineage of giant viruses brings a rhodopsin photosystem to unicellular marine predators. Proc Natl Acad Sci USA 116:20574–20583 31548428 10.1073/pnas.1907517116 PMC6789865

[CR97] Nicoll MP, Hann W, Shivkumar M, Harman LER, Connor V, Coleman HM, Proença JT, Efstathiou S (2016) The HSV-1 latency-associated transcript functions to repress latent phase lytic gene expression and suppress virus reactivation from latently infected neurons. PLOS Pathog 12:e1005539 27055281 10.1371/journal.ppat.1005539 PMC4824392

[CR98] Ogata H, Toyoda K, Tomaru Y, Nakayama N, Shirai Y, Claverie J-M, Nagasaki K (2009) Remarkable sequence similarity between the dinoflagellate-infecting marine girus and the terrestrial pathogen African swine fever virus. Virol J 6:178 19860921 10.1186/1743-422X-6-178 PMC2777158

[CR99] Philippe N, Legendre M, Doutre G, Couté Y, Poirot O, Lescot M, Arslan D, Seltzer V, Bertaux L, Bruley C et al (2013) Pandoraviruses: amoeba viruses with genomes up to 2.5 Mb reaching that of parasitic eukaryotes. Science 341:281–286 23869018 10.1126/science.1239181

[CR100] Pumplin N, Voinnet O (2013) RNA silencing suppression by plant pathogens: defence, counter-defence and counter-counter-defence. Nat Rev Microbiol 11:745–760 24129510 10.1038/nrmicro3120

[CR101] Raoult D, Audic S, Robert C, Abergel C, Renesto P, Ogata H, La Scola B, Suzan M, Claverie J-M (2004) The 1.2-megabase genome sequence of mimivirus. Science 306:1344–1350 15486256 10.1126/science.1101485

[CR102] Ratchinski P, Godfroy O, Noel B, Aury J-M, Cock JM (2025) Life-cycle-related gene expression patterns in the brown algae. eLife. 10.7554/eLife.107449.2 10.7554/eLife.107449 PMC12629598 41259243

[CR103] Raybaud V, Beaugrand G, Goberville E, Delebecq G, Destombe C, Valero M, Davoult D, Morin P, Gevaert F (2013) Decline in kelp in West Europe and climate. PLoS ONE 8:e66044 23840397 10.1371/journal.pone.0066044 PMC3694085

[CR104] Rigou S, Schmitt A, Moreno AB, Lartigue A, Danner L, Mayer L, Giry C, Trabelsi F, Belmudes L, Olivero-Deibe N et al (2025) Evolutionarily conserved grammar rules viral factories of amoeba-infecting members of the hyperdiverse Nucleocytoviricota phylum. Proc Natl Acad Sci USA 122:e2515074122 40864652 10.1073/pnas.2515074122 PMC12415211

[CR105] Ruiz Martínez E, Mckeown DA, Schroeder DC, Thuestad G, Sjøtun K, Sandaa R-A, Larsen A, Hoell IA (2023) Phaeoviruses present in cultured and natural kelp species, Saccharina latissima and Laminaria hyperborea (Phaeophyceae, Laminariales), in Norway. Viruses 15:2331 38140573 10.3390/v15122331 PMC10747701

[CR106] Sarre LA, Kim IV, Ovchinnikov V, Olivetta M, Suga H, Dudin O, Sebé-Pedrós A, de Mendoza A (2024) DNA methylation enables recurrent endogenization of giant viruses in an animal relative. Sci Adv 10:eado6406 38996012 10.1126/sciadv.ado6406 PMC11244446

[CR107] Sauvageau C (1896) Sur la nature des sporanges en chapelet de l’ectocarpus confervoides. J Bot 10:140–146

[CR108] Schroeder DC (2015) More to Phaeovirus infections than first meets the eye. Perspect Phycol 2:105–109

[CR109] Schulz F, Abergel C, Woyke T (2022) Giant virus biology and diversity in the era of genome-resolved metagenomics. Nat Rev Microbiol 20:721–736 35902763 10.1038/s41579-022-00754-5

[CR110] Scola BL, Audic S, Robert C, Jungang L, de Lamballerie X, Drancourt M, Birtles R, Claverie J-M, Raoult D (2003) A giant virus in amoebae. Science 299:2033 12663918 10.1126/science.1081867

[CR111] Sengco MR, Bräutigam M, Kapp M, Müller DG (1996) Detection of virus DNA in *Ectocarpus siliculosus* and *E. fasciculatus* (Phaeophyceae) from various geographic areas. Eur J Phycol 31:73–78

[CR112] Shaler TS, Fromm A, Schatz D, Ben-Dor S, Feldmesser E, Vardi A (2025) Mapping the transcriptional landscape of algal resistance to viral infection reveals a core expression program. New Phytol 248:1368–1384 40873210 10.1111/nph.70498 PMC12489294

[CR113] Shen Y, Motomura T, Ichihara K, Matsuda Y, Yoshimura K, Kosugi C, Nagasato C (2023) Application of CRISPR-Cas9 genome editing by microinjection of gametophytes of Saccharina japonica (Laminariales, Phaeophyceae). J Appl Phycol 35:1431–1441

[CR114] Sheng Y, Wu Z, Xu S, Wang Y (2022) Isolation and identification of a large green alga virus (*Chlorella* virus XW01) of *Mimiviridae* and its virophage (*Chlorella* virus virophage SW01) by using unicellular green algal cultures. J Virol 96:e02114–e02121 35262372 10.1128/jvi.02114-21 PMC9006914

[CR115] Steuerman Y, Cohen M, Peshes-Yaloz N, Valadarsky L, Cohn O, David E, Frishberg A, Mayo L, Bacharach E, Amit I et al (2018) Dissection of influenza infection in vivo by single-cell RNA sequencing. Cell Syst 6:679–691.e4 29886109 10.1016/j.cels.2018.05.008 PMC7185763

[CR116] Suskiewicz TS, Byrnes JEK, Steneck RS, Russell R, Wilson CJ, Rasher DB (2024) Ocean warming undermines the recovery resilience of New England kelp forests following a fishery-induced trophic cascade. Ecology 105:e4334 38887829 10.1002/ecy.4334

[CR117] Suttle CA (2005) Viruses in the sea. Nature 437:356–361 16163346 10.1038/nature04160

[CR118] Suzich JB, Cliffe AR (2018) Strength in diversity: understanding the pathways to herpes simplex virus reactivation. Virology 522:81–91 30014861 10.1016/j.virol.2018.07.011 PMC6092753

[CR119] Tang B, Feng L, Hulin MT, Ding P, Ma W (2023) Cell-type-specific responses to fungal infection in plants revealed by single-cell transcriptomics. Cell Host Microbe 31:1732–1747.e5 37741284 10.1016/j.chom.2023.08.019

[CR120] Terpis KX, Salomaki ED, Barcytė D, Pánek T, Verbruggen H, Kolisko M, Bailey JC, Eliáš M, Lane CE (2025) Multiple plastid losses within photosynthetic stramenopiles revealed by comprehensive phylogenomics. Curr Biol. 10.1016/j.cub.2024.11.065 10.1016/j.cub.2024.11.065 39793566

[CR121] Thomy J, Schvarcz CR, McBeain KA, Edwards KF, Steward GF (2024) Eukaryotic viruses encode the ribosomal protein eL40. Npj Viruses 2:1–11 39464202 10.1038/s44298-024-00060-2 PMC11499249

[CR122] Vieira HH, Bulzu P-A, Kasalický V, Haber M, Znachor P, Piwosz K, Ghai R (2024) Isolation of a widespread giant virus implicated in cryptophyte bloom collapse. ISME J 18:wrae029 38401169 10.1093/ismejo/wrae029 PMC10960955

[CR123] Vigneau J, Lotharukpong JS, Liu P, Luthringer R, Lombard B, Loew D, Haas FB, Borg M, Coelho SM (2025) Rewiring of chromatin regulation underlies the evolution of brown algal multicellularity. Preprint at 10.1101/2025.09.16.676480

[CR124] Vigneau J, Martinho C, Godfroy O, Zheng M, Haas FB, Borg M, Coelho SM (2024) Interactions between U and V sex chromosomes during the life cycle of Ectocarpus. Development 151:dev.202677 10.1242/dev.202677 PMC11057875 38512707

[CR125] Vincent F, Sheyn U, Porat Z, Schatz D, Vardi A (2021) Visualizing active viral infection reveals diverse cell fates in synchronized algal bloom demise. Proc Natl Acad Sci USA 118:e2021586118 33707211 10.1073/pnas.2021586118 PMC7980383

[CR126] Vincent F, Vardi A (2023) Viral infection in the ocean—a journey across scales. PLOS Biol 21:e3001966 36701270 10.1371/journal.pbio.3001966 PMC9879395

[CR127] Wernberg T, Thomsen MS, Tuya F, Kendrick GA, Staehr PA, Toohey BD (2010) Decreasing resilience of kelp beds along a latitudinal temperature gradient: potential implications for a warmer future. Ecol Lett 13:685–694 20412279 10.1111/j.1461-0248.2010.01466.x

[CR128] Williamson K, Eme L, Baños H, McCarthy CGP, Susko E, Kamikawa R, Orr RJS, Muñoz-Gómez SA, Minh BQ, Simpson AGB et al (2025) A robustly rooted tree of eukaryotes reveals their excavate ancestry. Nature 640:974–981 40074902 10.1038/s41586-025-08709-5

[CR129] Wilson WH, Van Etten JL, Allen MJ (2009) The phycodnaviridae: the story of how tiny giants rule the world. In: Van Etten JL (ed) Lesser known large DsDNA viruses. Springer, Berlin, pp 1–42 10.1007/978-3-540-68618-7_1 PMC2908299 19216434

[CR130] Zhang B, Zhou X, Ren Y (2025) Host-virus molecular arms race: RNAi-mediated antiviral defense and viral suppressor of RNAi. Cell Insight 4:100276 40994739 10.1016/j.cellin.2025.100276 PMC12454879

[CR131] Zhang M, Zhang T, Yu M, Chen Y-L, Jin M (2022) The life cycle transitions of temperate phages: regulating factors and potential ecological implications. Viruses 14:1904 36146712 10.3390/v14091904 PMC9502458

[CR132] Zhang Y, Jiang P, Gao J, Liao J, Sun S, Shen Z, Qin S (2008) Recombinant expression of rt-PA gene (encoding Reteplase) in gametophytes of the seaweed Laminaria japonica (Laminariales, Phaeophyta). Sci China C Life Sci 51:1116–1120 19093086 10.1007/s11427-008-0143-4

[CR133] Zhao H, Zhang R, Wu J, Meng L, Okazaki Y, Hikida H, Ogata H (2023) A 1.5-Mb continuous endogenous viral region in the arbuscular mycorrhizal fungus Rhizophagus irregularis. Virus Evol 9:vead064 37953976 10.1093/ve/vead064 PMC10640383

[CR134] Zhao J-H, Hua C-L, Fang Y-Y, Guo H-S (2016) The dual edge of RNA silencing suppressors in the virus–host interactions. Curr Opin Virol 17:39–44 26802204 10.1016/j.coviro.2015.12.002

[CR135] Zhu J, Moreno-Pérez A, Coaker G (2023) Understanding plant pathogen interactions using spatial and single-cell technologies. Commun Biol 6:814 37542114 10.1038/s42003-023-05156-8 PMC10403533

